# Cardiac arrest patients have an impaired immune response, which is not influenced by induced hypothermia

**DOI:** 10.1186/cc14002

**Published:** 2014-07-30

**Authors:** Charlotte J Beurskens, Janneke Horn, Anita M Tuip de Boer, Marcus J Schultz, Ester MM van Leeuwen, Margreeth B Vroom, Nicole P Juffermans

**Affiliations:** Laboratory of Experimental Intensive Care and Anaesthesiology, Academic Medical Center, University of Amsterdam, Meibergdreef 9, 1105 AZ Amsterdam, Netherlands; Department of Intensive Care, Academic Medical Center, University of Amsterdam, Meibergdreef 9, 1105 AZ Amsterdam, Netherlands; Department of Experimental Immunology, Academic Medical Center, University of Amsterdam, Meibergdreef 9, 1105 AZ Amsterdam, Netherlands; Laboratory of Experimental Intensive Care and Anaesthesiology (L.E.I.C.A.) / Department of Intensive Care Medicine, Academic Medical Center, Room M0-210, Meibergdreef 9, 1105 AZ Amsterdam, Netherlands

## Abstract

**Introduction:**

Induced hypothermia is increasingly applied as a therapeutic intervention in ICUs. One of the underlying mechanisms of the beneficial effects of hypothermia is proposed to be reduction of the inflammatory response. However, a fear of reducing the inflammatory response is an increased infection risk. Therefore, we studied the effect of induced hypothermia on immune response after cardiac arrest.

**Methods:**

A prospective observational cohort study in a mixed surgical-medical ICU. Patients admitted at the ICU after surviving cardiac arrest were included and during 24 hours body temperature was strictly regulated at 33°C or 36°C. Blood was drawn at three time points: after reaching target temperature, at the end of the target temperature protocol and after rewarming to 37°C. Plasma cytokine levels and response of blood leucocytes to stimulation with toll-like receptor (TLR) ligands lipopolysaccharide (LPS) from Gram-negative bacteria and lipoteicoic acid (LTA) from Gram-positive bacteria were measured. Also, monocyte HLA-DR expression was determined.

**Results:**

In total, 20 patients were enrolled in the study. Compared to healthy controls, cardiac arrest patients kept at 36°C (n = 9) had increased plasma cytokines levels, which was not apparent in patients kept at 33°C (n = 11). Immune response to TLR ligands in patients after cardiac arrest was generally reduced and associated with lower HLA-DR expression. Patients kept at 33°C had preserved ability of immune cells to respond to LPS and LTA compared to patients kept at 36°C. These differences disappeared over time. HLA-DR expression did not differ between 33°C and 36°C.

**Conclusions:**

Patients after cardiac arrest have a modest systemic inflammatory response compared to healthy controls, associated with lower HLA-DR expression and attenuated immune response to Gram-negative and Gram-positive antigens, the latter indicative of an impaired immune response to bacteria. Patients with a body temperature of 33°C did not differ from patients with a body temperature of 36°C, suggesting induced hypothermia does not affect immune response in patients with cardiac arrest.

**Trial registration:**

ClinicalTrials.gov NCT01020916, registered 25 November 2009

## Introduction

Induced hypothermia is applied clinically to reduce ischemia-reperfusion injury during operative procedures and following cardiac arrest [1–3]. In cardiopulmonary surgery, hypothermia is associated with improved neurological outcome [4, 5]. Also, avoiding hyperthermia by controlling body temperature is associated with a favourable neurologic function in survivors of cardiac arrest [6], as well as with earlier shock reversal in septic shock patients [7]. Inhibition of the exaggerated systemic inflammatory response syndrome (SIRS) is thought to be one of the mechanisms through which temperature management can mitigate the harmful effects of ischemia-reperfusion. In a pig model of cardiac arrest, hypothermia reduced expression of pro-inflammatory cytokines within the brain [8]. Also in other models of hyper-inflammatory conditions, hypothermia reduced organ failure associated with a decrease of the inflammatory response [9–15]. Also in infectious disease models, hypothermia was associated with a reduction in organ failure [9, 16]. Thereby, hypothermia may mitigate harm caused by an ‘overshoot’ of a systemic inflammatory response.

On the other hand, an adequate adaptive host immune response to pathogens and infection is crucial [17]. Fever has long been considered an important factor for optimal antimicrobial host defense, [18–21].

Hypothermia inhibits immune response [22], with delayed generation of pro-inflammatory cytokines by monocytes [23] and reduction of neutrophil and monocyte migration [24, 25]. The consequence of inhibiting host immune response by induced hypothermia may be a higher infection risk. In particular, patients shortly after a cardiac arrest suffer from a dysregulated production of cytokines and may therefore be susceptible to nosocomial infection [26]. Two randomized trials in cardiac arrest patients reported no increased overall infection rates associated with hypothermia [1, 27], although a trend towards more infection could be noted [27]. Also trials in traumatic brain injury have not reported increased infection rate following hypothermia [28, 29], although not all studies are consistent [30]. In addition, prolonged hypothermia (for more than 48 hours) did not increase risk of infection in patients with brain injury [31–33], taking into account that some of these patients received selective decontamination of the digestive tract [32].

However, a recent systematic review in patients enrolled in randomized controlled clinical trials of therapeutic hypothermia for any indication, showed an association of hypothermia with increased prevalence of pneumonia and sepsis, although overall infection rate was not affected [34]. Taken together, the effect of hypothermia on the risk of infections is unclear. Patient studies describing the effect of induced hypothermia specifically on the innate immune response are scarce and studies lack an adequate control group [35]. Recently, the Temperature Target Management (TTM) trial was concluded, in which cardiac arrest patients were randomized between maintaining body temperature at 33°C or at 36°C [27]. In this predefined substudy of the TTM trial, we investigated the effect of induced hypothermia on the innate immune response to toll-like receptor (TLR) ligands. In humans, TLRs are critical in the first host immune response to pathogens by mediating cytokine secretion [36]. In addition, we measured human leukocyte antigen-DR (HLA-DR) surface expression on monocytes, as low HLA-DR expression is associated with secondary infection and mortality in critically ill patients [37, 38] and hypothermia was shown to reduce HLA-DR expression *in vitro*[39]. We hypothesized that SIRS is reduced in patients with a target temperature of 33°C compared to patients with a target temperature of 36°C, but that hypothermia does not affect the immune response to pathogen-associated molecular patterns (lipopolysaccharide (LPS) and lipoteicoic acid (LTA)).

## Methods

### Patient inclusion

The study was approved by the local medical ethics committee of the Academic Medical Center, University of Amsterdam, the Netherlands (NL32522.018.10) and conducted in concordance with the principles of the Declaration of Helsinki and good clinical practice. From January 2011 until October 2012, adult patients admitted to the mixed surgical-medical intensive care unit (ICU) of a tertiary referral center in Amsterdam, the Netherlands after out-of-hospital cardiac arrest with a Glasgow Coma Score <8 and treated with therapeutic hypothermia (33°C) for 24 hours, were included in our study after their relatives gave informed consent. From March 2011, our center started enrollment in the TTM trial, patients who enrolled in the TTM trial were only included for our substudy after additional informed consent was obtained from the relatives. Exclusion criteria were pregnancy, out-of-hospital cardiac arrest of presumed non-cardiac cause, in-hospital cardiac arrest, known bleeding diathesis, suspected or confirmed acute intracranial bleeding, suspected or confirmed acute stroke, temperature on admission <30°C, unwitnessed asystole, persistent cardiogenic shock, known limitations in therapy, known disease making 180-day survival unlikely, known pre-arrest cerebral performance category 3 or 4, >240 minutes from return of spontaneous circulation (ROSC) to randomisation [27]. Patients included prior to the TTM trial (n = 8; target temperature 32 to 34°C) and during the TTM trial (n = 12, 3 in the 33°C group and 9 in the 36°C group) did not differ in patients characteristics, Acute Physiology and Chronic Health Evaluation (APACHE) III, Simplified Acute Physiology Score (SAPS) II score, time to ROSC and cause of cardiac arrest (data not shown). A total of 20 patients were included, of whom 11 with a target temperature of 33°C and 9 patients with a target temperature of 36°C. Healthy volunteers were recruited and included after informed consent for a single blood donation (n = 4, mean age 28 years, 75% female).

### Study procedure

Patients included in our study received standard post-resuscitation care according to the current best practice or the post-resuscitation protocol of the TTM trial [27], including 24 hours of target temperature management to achieve a core body temperature of either 33°C or 36°C with the use of ice-cold saline (maximum 1 L) and a cooling device (Blanket roll, Cincinnatti Sub-Zero, Cincinnatti, OH, USA). Temperature was measured using a bladder catheter. All patients were sedated with propofol, mechanically ventilated in a pressure-controlled mode and selective digestive tract decontamination was administered by intestinal and oropharyngeal application of topical, non-absorbable antibiotics (100 mg polymyxine E, 80 mg tobramycine, 500 mg amphothericine B) during the whole ICU stay in combination with intravenous administration of cefotaxime during the first four days of ICU stay. Patients received either anticoagulant therapy as deemed appropriate or thrombosis prophylaxis. Patients were not fed. Blood was drawn at three time points: after reaching target temperature (33°C or 36°C; T = 1), at the end of the target temperature protocol (T = 2) and after reaching 37°C (T = 3). Healthy volunteers donated only once.

### Measurements and data collection

Data from the patient data monitoring system were collected, including previous medical history, age, gender, weight, length, maximal leukocyte count, APACHE III, SAPS II score, as registered in the Dutch National Intensive Care Evaluation [40].

Serum levels of interleukin (IL)-1β, IL-1RA, IL-8, IL-10, macrophage inflammatory proteins (MIP)-1, monocyte chemotactic protein (MCP)-1 and soluble CD40 ligand were determined by Luminex, according to the manufacturer’s instructions (Merck Millipore Chemicals BV, Amsterdam, the Netherlands). Serum levels of IL-6 and tumor necrosis factor (TNF)-α were determined by enzyme-linked immunosorbent assay (ELISA), according to the manufacturer’s instructions (R&D Systems, Abingdon, United Kingdom).

### Whole blood stimulation

The response of blood leucocytes to stimulation with TLR ligands was determined in a whole blood stimulation system. Immediately after drawing, blood was diluted 1:1 with RPMI and stimulated with LPS (100 ng/ml; Sigma-Aldrich, Steinheim, Germany) or LTA (10 μg/ml; Invivogen, San Diego, CA, USA) as bacterial antigens of respectively Gram-negative and Gram-positive bacteria. After 2 or 24 hours stimulation in a 37°C incubator with 5% CO_2_, whole blood was centrifuged at 600 g for 10 minutes at 4°C. Supernatant was stored at −80°C and levels of IL-6 and TNF-α were determined by ELISA, according to the manufacturer’s instructions (R&D Systems).

### HLA-DR expression

HLA-DR expression on monocytes was analysed by fluorescence-activated cell sorter after labelling by incubation with HLA-DR-FITC and CD14-PE monoclonal antibodies (Becton Dickinson, Erembogem, Belgium). Erythrocytes were lysed with lysis buffer (Becton Dickinson BV, Breda, the Netherlands) and the debris was washed away. The remaining leukocytes were fixated with 1% paraformaldehyde. The final flow cytometric analysis was done, using a flow cytometry (Becton Dickinson BV, Breda, the Netherlands). HLA-DR expression was measured on CD14-bright monocytes, which have mainly anti-inflammatory properties [41] and CD14-dull monocytes, which are more pro-inflammatory, since they selectively induce production of cytokines in response to viruses and immune complexes containing nucleic acids [42]. To compare patient samples measured at different time points, a standard series of quantum beads, which are labelled with a known quantity of FITC fluorescent label (Quantum FITC-5 MESF (Premix), Bangs Laboratories, Fishers, IN, USA) was analysed simultaneously with each measurement. Therefore, HLA-DR expression could be expressed in molecules of equivalent soluble fluorochrome (MESF) units.

### Statistical analysis

Data are expressed by mean ± standard deviation (SD) in the table and as median ± range in the figures, depending on distribution of the data. Differences between the 33°C and 36°C groups were compared using an unpaired *t* test or a Mann-Whitney *U* test, depending on distribution of the data. The effect of temperature over time was compared using a repeated measurement analysis of variance (ANOVA) or a Friedman test with either a Bonferroni's or Dunn's multiple comparison test correction. Differences between healthy volunteers and patients with a target temperature of either 33°C or 36°C over time were compared using a one-way ANOVA or Kruskal-Wallis test, with either a Bonferroni's or Dunn's multiple comparison test, depending on distribution of the data. Statistical significance was set at *P* <0.05.

## Results

### Patient characteristics

Patients in the 33°C and 36°C groups did not differ in previous medical history or disease severity (Table 1). After reaching target temperature at T = 1, body temperature was 33.2 ± 0.7°C in patients with a target temperature of 33°C and 35.6 ± 0.9°C in patients with a target temperature of 36°C (*P* = 0.002). At T = 1, plasma samples could not be collected from four patients. One patient with a target temperature of 33°C died before the last time point of blood sampling (T = 3). A confirmed infection within 24 hours after ICU admittance occurred in two patients, one patient in each group (Table 1).

**Table 1:** **Baseline characteristics of 11 patients with a target temperature of 33°C compared 9 patients with a target temperature of 36°C, after reaching target temperature**

	33°C	36°C	***P*** value
***Gender (male/female)***	8/3	8/1	
***Age (years)***	64 ± 15	61 ± 15	0.70
***Length (cm)***	173 ± 12	178 ± 7	0.27
***Weight (kg)***	81 ± 24	81 ± 13	0.95
***Maximal leukocyte count***	15.8 ± 4.9	16.1 ± 4.0	0.92
***SAPS II score***	54 ± 18	59 ± 18	0.58
***APACHE III score***	80 ± 44	88 ± 34	0.71
***Previous medical history***			
Chronic cardiovascular insufficiency	1	0	
Arrhythmia	1	0	
Cerebrovascular accident	1	0	
Chronic kidney failure	0	0	
Diabetes	1	1	
COPD	1	0	
Chronic respiratory failure	0	0	
Cirrhosis	0	0	
AIDS	0	0	
Immune insufficiency	0	0	
Malignancy	0	0	

Data expressed as mean ± SD. APACHE: Acute Physiology and Chronic Health Evaluation; SAPS: Simplified Acute Physiology Score; COPD: chronic obstructive pulmonary disease.

### The effect of hypothermia on the SIRS reaction

**Figure 1:**
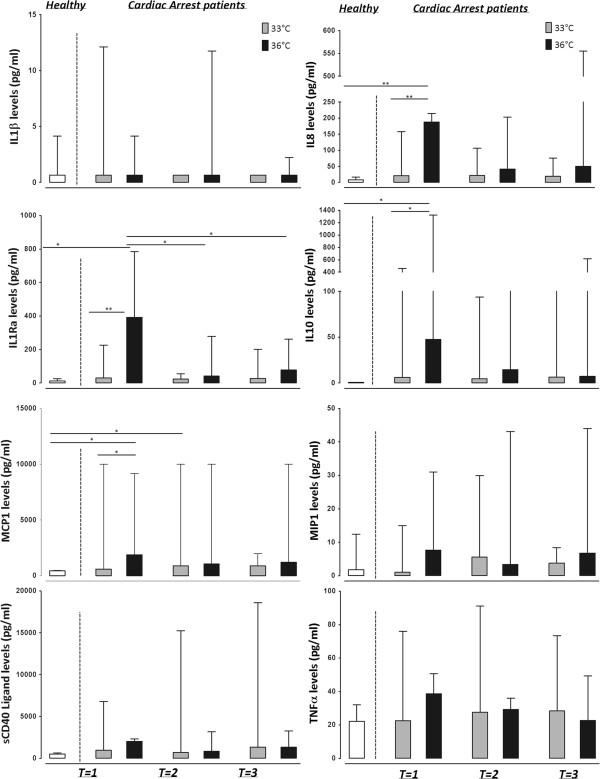
**Cytokine levels in plasma of healthy controls, cardiac arrest patients with a target temperature of 33°C and cardiac arrest patients with a target temperature of 36°C, after reaching target temperature (T = 1), at the end of the target temperature protocol (T = 2) and after reaching 37°C (T = 3).**

Baseline inflammatory response was measured in plasma cytokines levels (Figure 1). Levels of IL-1RA, IL-8, IL-10 and MCP-1 showed an increase in the patient group with a target temperature of 36°C compared to healthy controls, which was not apparent in the 33°C group. Levels of IL-1RA, but not of other cytokines, decreased after 24 hours of temperature management protocol (T = 1 vs. T = 2; Figure 1) in the group with a target temperature of 36°C. Levels of IL-1RA, IL-8, IL-10 and MCP-1 were higher in the 36°C group compared to the 33°C group shortly after reaching target temperature, but these differences disappeared during temperature management over time (T = 1; Figure 1). Plasma levels of IL-1β, MIP-1, soluble CD40 ligand and TNF-α levels were not increased in patients compared to healthy controls, nor were there differences between the 33°C group and the 36°C group.

### The effect of hypothermia on the immune response to TLR ligands

**Figure 2:**
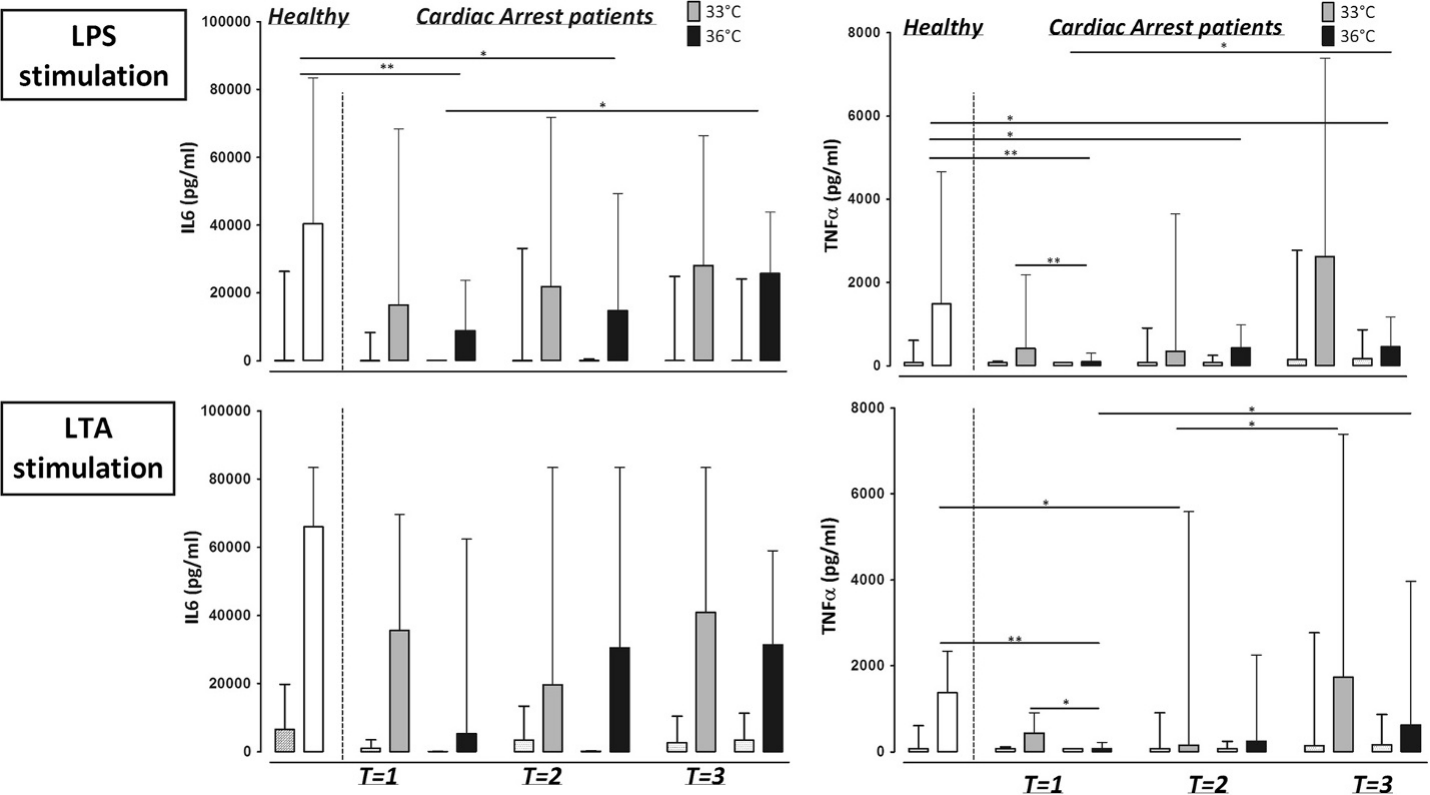
**Cytokine production after whole blood stimulation with LPS or LTA, in plasma of healthy controls, cardiac arrest patients with a target temperature of 33°C and cardiac arrest patients with a target temperature of 36°C, after reaching target temperature (T = 1), at the end of the target temperature protocol (T = 2) and after reaching 37°C (T = 3)**. Healthy volunteers are marked by white bars; cardiac arrest patients with a target temperature of 33°C are marked by grey bars, cardiac arrest patients with a target temperature of 36°C are marked by black bars. Every measurement is preceded by a blank control stimulation. Data expressed as median with range. *P <0.05; **P <0.01. LPS: lipopolysaccharide; LTA: lipoteicoic acid.

Results of whole blood stimulated for two hours were not different from 24 hours stimulation. Therefore, only the results of 24 hours stimulation are shown (Figure 2).

#### Response to LPS

The immune response to stimulation with Gram-negative antigen LPS resulted in lower levels of IL-6 and TNF-α in patients of the 36°C group compared to healthy controls, which increased towards the end of the temperature management protocol in the group with a target temperature of 36°C (Figure 2, upper panel; T = 1 vs. T = 3). This increase was not observed in the 33°C group. TNF-α levels were decreased in the 36°C group compared to the 33°C group at baseline. However, no significant differences between the groups appeared during the 24 hours of temperature management and after rewarming.

#### Response to LTA

Production of IL-6 did not differ between patient groups and healthy controls, nor were there differences over time between the 33°C and 36°C groups (Figure 2, lower panel). The TNF production in response to stimulation with the Gram-positive antigen LTA was decreased in patients with a target temperature of 36°C compared to healthy controls at the start of the temperature management and increased again after regaining normothermia (T = 1 vs. T = 3; Figure 2). TNF-α level was also lower in the 36°C group compared to the 33°C group at T = 1, but this difference between groups disappeared during temperature management over time.

### The effect of hypothermia on HLA-DR expression

**Figure 3:**
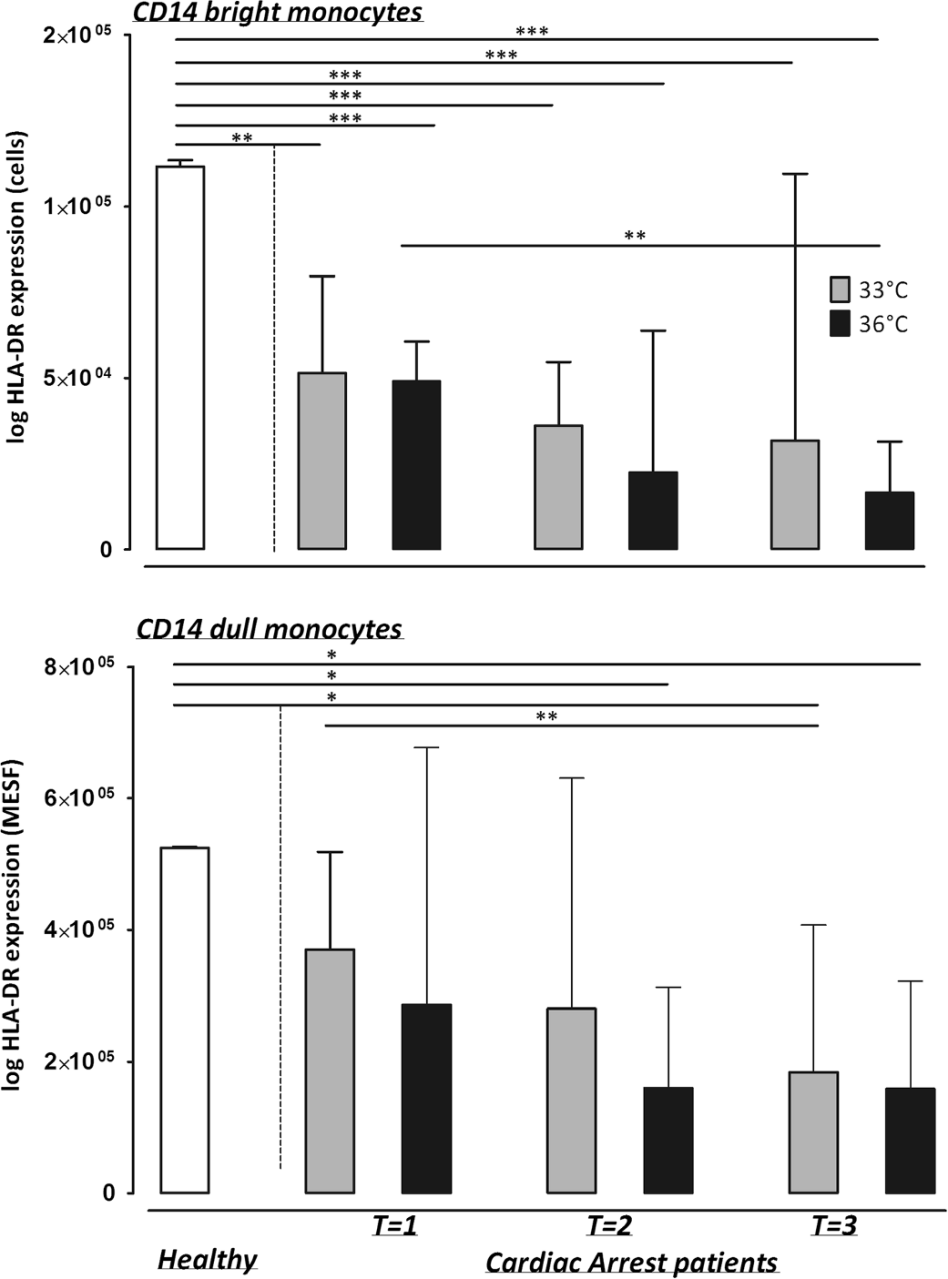
**HLA-DR expression on bright and dull monocytes in plasma of healthy controls, cardiac arrest patients with a target temperature of 33°C and cardiac arrest patients with a target temperature of 36°C, after reaching target temperature (T = 1), at the end of the target temperature protocol (T = 2) and after reaching 37°C (T = 3).** HLA-DR: human leukocyte antigen-DR.

HLA-DR expression on CD14-bright monocytes was reduced in patients compared to healthy controls at all time points for both the 33°C and 36°C group (Figure 3). HLA-DR expression on both bright and dull monocytes further decreased over time (T = 1 vs. T = 3; Figure 3). HLA-DR expression showed no differences between the 33°C and 36°C groups.

## Discussion

Patients after cardiac arrest demonstrate a SIRS compared to healthy controls, which was most noted for those who were kept at 36°C and blunted for those who were cooled to 33°C. This SIRS reaction was associated with lower HLA-DR expression and an attenuated immune response to Gram-negative and Gram-positive antigens compared to healthy controls. Between patients with a targeted body temperature of 33°C compared to 36°C, there were no differences in immune response to the bacterial antigens.

An increased systemic inflammatory response following cardiac arrest compared to healthy controls has been shown before [26]. The inflammatory response in cardiac arrest patients treated with induced hypothermia, was previously studied by Bisschops *et al*., showing a temporary increase of IL-6 levels during hypothermia and increased levels of IL-8 and MCP-1 compared to baseline [35], with levels largely comparable to our patients. Anti-inflammatory cytokines IL-1RA and IL-10 were unaltered compared to baseline [35]. However, in this study, effects of body temperature could not be dissected from effects of ischemia-reperfusion injury following cardiac arrest. Our study expands on these findings by including a normothermic control group over time. We found that initial cytokine levels were reduced in patients with a target temperature of 33°C compared to 36°C, although differences were small. This decrease in inflammatory response is in line with multiple experimental studies demonstrating an inhibitory effect of hypothermia on cytokine levels [10–13, 23]. However, at the end of the 24 hours of the temperature management protocol, there were no differences between both groups in plasma cytokine levels. Thereby, any effect of hypothermia on systemic inflammatory response seems temporary.

The systemic inflammatory response in patients was accompanied by an initial decreased ability of immune cells to respond to TLR ligands LPS and LTA, although not all cytokines were affected. The ability to generate an immune response can also be measured by HLA-DR expression. Of interest, we found a clear reduction in the expression of HLA-DR following cardiac arrest, compared to healthy controls. A blunted host immune response to TLR ligands and a decreased HLA-DR expression have been shown before in septic shock patients [38, 43], which is thought to contribute to an increased risk of nosocomial infection. To the best of our knowledge, this attenuated immune response to bacterial antigens after cardiac arrest has not been reported before. Decreased HLA-DR expression together with a blunted response to TLR ligands suggests that also cardiac arrest patients have an impaired immune response to pathogens. In line with this, nosocomial infection occurs in up to 50 to 65% of cardiac arrest patients [44–46]. Thereby, cardiac arrest patients should be closely monitored for the development of nosocomial infection. Whether prophylactic antibiotics are warranted in this patient group remains to be determined.

To investigate effects of induced hypothermia on immune response in cardiac arrest patients, our study provided an appropriate experimental setting in a more or less homogenous patient population. We found that the decreased ability of immune cells to produce TNF-α in response to TLR ligands was more prominent in patients with a target temperature of 36°C compared to 33°C. Thereby, hypothermia does not seem to have an inhibitory effect on immune response to TLR ligands compared to normothermia. Also, we found no differences in HLA-DR expression between the 33°C and 36°C groups at any time points. These results are in line with the TTM trial, which found no significant differences in infection rate between the 33°C and 36°C groups in a large cohort of cardiac arrest patients [27]. In contrast, a previous study in cardiac arrest patients found that induced hypothermia was an independent risk factor for infection [44]. However, that study lacked a normothermic control group. Also, a meta-analysis suggests that hypothermia increases risk of infection, but in a very heterogeneous patient population [34]. Taken together, our results suggest that having had a cardiac arrest may render the patient susceptible to infection by a decreased immune response, but this risk of infection is not further increased by induced (mild) hypothermia. Whether the immune response is hampered at temperatures below 33°C cannot be dissected from this study [47].

Limitations of our study are small sample size. However, larger sample sizes with these kinds of investigations including whole blood stimulations are intricate, since they are labour-intensive and expensive. However, our sample size is comparable to previous studies [32, 35]. Also, healthy controls were not age-matched to patients, which may have affected the immune response. Lastly, the inclusion period was extensive, but our standard of care protocol did not undergo relevant changes during this time period.

The relevance of our findings is that lowering body temperature may be done safely without compromising the immune response in patient groups who are thought to benefit from regulating body temperature. This may be especially interesting for those patients who suffer from an increased inflammatory response. Currently, a large trial on hypothermia in sepsis patients is ongoing (Trial number: NCT01455116).

## Conclusions

Patients after cardiac arrest have a modest systemic inflammatory response compared to healthy controls. Cardiac arrest was associated with lower HLA-DR expression and attenuated immune response to Gram-negative and Gram-positive antigens, indicative of an impaired immune response. A body temperature of 33°C did not influence immune reaction compared to 36°C, suggesting that induced hypothermia in itself does not affect immune response.

## Key messages

- ● Cardiac arrest patients have a decreased systemic inflammatory response compared to healthy controls
- ● However, hypothermia to 33°C for 24 hours does not further affect the immune response, compared to 36°C

## Abbreviations

APACHE: Acute Physiology and Chronic Health Evaluation
ELISA: enzyme-linked immunosorbent assay
HLA-DR: human leukocyte antigen-DR
ICU: intensive care unit
IL: interleukin
LPS: lipopolysaccharide
LTA: lipoteicoic acid
MCP: monocyte chemotactic protein
MESF: molecules of equivalent soluble fluorochrome
MIP: macrophage inflammatory proteins
ROSC: return of spontaneous circulation
SAPS: Simplified Acute Physiology Score
SIRS: systemic inflammatory response syndrome
TNF-α: tumor necrosis factor
TLR: toll-like receptor
TTM: Temperature Target Management.

## References

[1] **Mild therapeutic hypothermia to improve the neurologic outcome after cardiac arrest** *N Engl J Med* 2002, **346:**549–556. 10.1056/NEJMoa012689 11856793

[2] Nolan JP, Deakin CD, Soar J, Bottiger BW, Smith G, European Resuscitation C (2005). European Resuscitation Council guidelines for resuscitation 2005. Section 4. Adult advanced life support. Resuscitation.

[3] Rees K, Beranek-Stanley M, Burke M, Ebrahim S (2001). Hypothermia to reduce neurological damage following coronary artery bypass surgery. Cochrane Database Syst Rev.

[4] Rao V, Christakis GT, Weisel RD, Ivanov J, Peniston CM, Ikonomidis JS, Shirai T (1995). Risk factors for stroke following coronary bypass surgery. J Card Surg.

[5] Martin TD, Craver JM, Gott JP, Weintraub WS, Ramsay J, Mora CT, Guyton RA (1994). Prospective, randomized trial of retrograde warm blood cardioplegia: myocardial benefit and neurologic threat. Ann Thorac Surg.

[6] Zeiner A, Holzer M, Sterz F, Schörkhuber W, Eisenburger P, Havel C, Kliegel A, Laggner AN (2001). Hyperthermia after cardiac arrest is associated with an unfavorable neurologic outcome. Arch Intern Med.

[7] Schortgen F, Clabault K, Katsahian S, Devaquet J, Mercat A, Deye N, Dellamonica J, Bouadma L, Cook F, Beji O, Brun-Buisson C, Lemaire F (2012). Fever control using external cooling in septic shock: a randomized controlled trial. Am J Respir Crit Care Med.

[8] Meybohm P, Gruenewald M, Zacharowski KD, Albrecht M, Lucius R, Fosel N, Hensler J, Zitta K, Bein B (2010). Mild hypothermia alone or in combination with anesthetic post-conditioning reduces expression of inflammatory cytokines in the cerebral cortex of pigs after cardiopulmonary resuscitation. Crit Care.

[9] Sarcia PJ, Scumpia PO, Moldawer LL, DeMarco VG, Skimming JW (2003). Hypothermia induces interleukin-10 and attenuates injury in the lungs of endotoxemic rats. Shock.

[10] Beurskens CJ, Aslami H, Kuipers MT, Horn J, Vroom MB, van Kuilenburg AB, Roelofs JJ, Schultz MJ, Juffermans NP (2012). Induced hypothermia is protective in a rat model of pneumococcal pneumonia associated with increased adenosine triphosphate availability and turnover. Crit Care Med.

[11] Lim CM, Kim MS, Ahn JJ, Kim MJ, Kwon Y, Lee I, Koh Y, Kim DS, Kim WD (2003). Hypothermia protects against endotoxin-induced acute lung injury in rats. Intensive Care Med.

[12] Taniguchi T, Kanakura H, Takemoto Y, Yamamoto K (2003). Effects of hypothermia on mortality and inflammatory responses to endotoxin-induced shock in rats. Clin Diagn Lab Immunol.

[13] Chu SJ, Perng WC, Hung CM, Chang DM, Lin SH, Huang KL (2005). Effects of various body temperatures after lipopolysaccharide-induced lung injury in rats. Chest.

[14] Chin JY, Koh Y, Kim MJ, Kim HS, Kim WS, Kim DS, Kim WD, Lim CM (2007). The effects of hypothermia on endotoxin-primed lung. Anesth Analg.

[15] Kira S, Daa T, Kashima K, Mori M, Noguchi T, Yokoyama S (2005). Mild hypothermia reduces expression of intercellular adhesion molecule-1 (ICAM-1) and the accumulation of neutrophils after acid-induced lung injury in the rat. Acta Anaesthesiol Scand.

[16] L'Her E, Amerand A, Vettier A, Sebert P (2006). Effects of mild induced hypothermia during experimental sepsis. Crit Care Med.

[17] Kluger MJ, Kozak W, Conn CA, Leon LR, Soszynski D (1996). The adaptive value of fever. Infect Dis Clin North Am.

[18] Jiang Q, Cross AS, Singh IS, Chen TT, Viscardi RM, Hasday JD (2000). Febrile core temperature is essential for optimal host defense in bacterial peritonitis. Infect Immun.

[19] Torossian A, Ruehlmann S, Middeke M, Sessler DI, Lorenz W, Wulf HF, Bauhofer A (2004). Mild preseptic hypothermia is detrimental in rats. Crit Care Med.

[20] Su F, Nguyen ND, Wang Z, Cai Y, Rogiers P, Vincent JL (2005). Fever control in septic shock: beneficial or harmful?. Shock.

[21] Bernheim HA, Kluger MJ (1976). Fever: effect of drug-induced antipyresis on survival. Science.

[22] Klastersky J, Kass EH (1970). Is suppression of fever or hypothermia useful in experimental and clinical infectious diseases?. J Infect Dis.

[23] Kimura A, Sakurada S, Ohkuni H, Todome Y, Kurata K (2002). Moderate hypothermia delays proinflammatory cytokine production of human peripheral blood mononuclear cells. Crit Care Med.

[24] Biggar WD, Bohn DJ, Kent G, Barker C, Hamilton G (1984). Neutrophil migration in vitro and in vivo during hypothermia. Infect Immun.

[25] Biggar WD, Barker C, Bohn D, Kent G (1986). Partial recovery of neutrophil functions during prolonged hypothermia in pigs. J Appl Physiol (1985).

[26] Adrie C, Adib-Conquy M, Laurent I, Monchi M, Vinsonneau C, Fitting C, Fraisse F, Dinh-Xuan AT, Carli P, Spaulding C, Dhainaut JF, Cavaillon JM (2002). Successful cardiopulmonary resuscitation after cardiac arrest as a "sepsis-like" syndrome. Circulation.

[27] Nielsen N, Wetterslev J, Cronberg T, Erlinge D, Gasche Y, Hassager C, Horn J, Hovdenes J, Kjaergaard J, Kuiper M, Pellis T, Stammet P, Wanscher M, Wise MP, Aneman A, Al-Subaie N, Boesgaard S, Bro-Jeppesen J, Brunetti I, Bugge JF, Hingston CD, Juffermans NP, Koopmans M, Kober L, Langorgen J, Lilja G, Moller JE, Rundgren M, Rylander C, Smid O, Werer C, Winkel P, Friberg H (2013). Targeted temperature management at 33 degrees C versus 36 degrees C after cardiac arrest. N Engl J Med.

[28] Clifton GL, Miller ER, Choi SC, Levin HS, McCauley S, Smith KR, Muizelaar JP, Wagner FC, Marion DW, Luerssen TG, Chesnut RM, Schwartz M (2001). Lack of effect of induction of hypothermia after acute brain injury. N Engl J Med.

[29] Clifton GL, Valadka A, Zygun D, Coffey CS, Drever P, Fourwinds S, Janis LS, Wilde E, Taylor P, Harshman K, Conley A, Puccio A, Levin HS, McCauley SR, Bucholz RD, Smith KR, Schmidt JH, Scott JN, Yonas H, Okonkwo DO (2011). Very early hypothermia induction in patients with severe brain injury (the National Acute Brain Injury Study: Hypothermia II): a randomised trial. Lancet Neurol.

[30] Qiu W, Zhang Y, Sheng H, Zhang J, Wang W, Liu W, Chen K, Zhou J, Xu Z (2007). Effects of therapeutic mild hypothermia on patients with severe traumatic brain injury after craniotomy. J Crit Care.

[31] Polderman KH, Tjong Tjin Joe R, Peerdeman SM, Vandertop WP, Girbes AR (2002). Effects of therapeutic hypothermia on intracranial pressure and outcome in patients with severe head injury. Intensive Care Med.

[32] Kamps M, Bisschops L, van der Hoeven JG, Hoedemaekers CW (2011). Hypothermia does not increase the risk of infection: a case control study. Crit Care.

[33] Qiu W, Shen H, Zhang Y, Wang W, Liu W, Jiang Q, Luo M, Manou M (2006). Noninvasive selective brain cooling by head and neck cooling is protective in severe traumatic brain injury. J Clin Neurosci.

[34] Geurts M, Macleod MR, Kollmar R, Kremer PH, van der Worp HB (2014). Therapeutic hypothermia and the risk of infection: a systematic review and meta-analysis. Crit Care Med.

[35] Bisschops LL, Hoedemaekers CW, Mollnes TE, van der Hoeven JG (2012). Rewarming after hypothermia after cardiac arrest shifts the inflammatory balance. Crit Care Med.

[36] Bekeredjian-Ding I, Jego G (2009). Toll-like receptors–sentries in the B-cell response. Immunology.

[37] Monneret G, Elmenkouri N, Bohe J, Debard AL, Gutowski MC, Bienvenu J, Lepape A (2002). Analytical requirements for measuring monocytic human lymphocyte antigen DR by flow cytometry: application to the monitoring of patients with septic shock. Clin Chem.

[38] Lukaszewicz AC, Grienay M, Resche-Rigon M, Pirracchio R, Faivre V, Boval B, Payen D (2009). Monocytic HLA-DR expression in intensive care patients: interest for prognosis and secondary infection prediction. Crit Care Med.

[39] Qadan M, Gardner SA, Vitale DS, Lominadze D, Joshua IG, Polk HC (2009). Hypothermia and surgery: immunologic mechanisms for current practice. Ann Surg.

[40] Arts D, de Keizer N, Scheffer GJ, de Jonge E (2002). Quality of data collected for severity of illness scores in the Dutch National Intensive Care Evaluation (NICE) registry. Intensive Care Med.

[41] Skrzeczynska-Moncznik J, Bzowska M, Loseke S, Grage-Griebenow E, Zembala M, Pryjma J (2008). Peripheral blood CD14high CD16+ monocytes are main producers of IL-10. Scand J Immunol.

[42] Cros J, Cagnard N, Woollard K, Patey N, Zhang SY, Senechal B, Puel A, Biswas SK, Moshous D, Picard C, Jais JP, D'Cruz D, Casanova JL, Trouillet C, Geissmann F (2010). Human CD14dim monocytes patrol and sense nucleic acids and viruses via TLR7 and TLR8 receptors. Immunity.

[43] Fumeaux T, Pugin J (2002). Role of interleukin-10 in the intracellular sequestration of human leukocyte antigen-DR in monocytes during septic shock. Am J Respir Crit Care Med.

[44] Perbet S, Mongardon N, Dumas F, Bruel C, Lemiale V, Mourvillier B, Carli P, Varenne O, Mira JP, Wolff M, Cariou A (2011). Early-onset pneumonia after cardiac arrest: characteristics, risk factors and influence on prognosis. Am J Respir Crit Care Med.

[45] Rello J, Valles J, Jubert P, Ferrer A, Domingo C, Mariscal D, Fontanals D, Artigas A (1995). Lower respiratory tract infections following cardiac arrest and cardiopulmonary resuscitation. Clin Infect Dis.

[46] Tsai MS, Chiang WC, Lee CC, Hsieh CC, Ko PC, Hsu CY, Su CP, Chen SY, Chang WT, Yuan A, Ma MH, Chen SC, Chen WJ (2005). Infections in the survivors of out-of-hospital cardiac arrest in the first 7 days. Intensive Care Med.

[47] Polderman KH, Herold I (2009). Therapeutic hypothermia and controlled normothermia in the intensive care unit: practical considerations, side effects, and cooling methods. Crit Care Med.

